# Development and Validation of a Scoring System for Hemorrhage Risk in Brain Arteriovenous Malformations

**DOI:** 10.1001/jamanetworkopen.2023.1070

**Published:** 2023-03-01

**Authors:** Yu Chen, Heze Han, Xiangyu Meng, Hengwei Jin, Dezhi Gao, Li Ma, Ruinan Li, Zhipeng Li, Debin Yan, Haibin Zhang, Kexin Yuan, Ke Wang, Yukun Zhang, Yang Zhao, Weitao Jin, Runting Li, Fa Lin, Xiaofeng Chao, Zhengfeng Lin, Qiang Hao, Hao Wang, Xun Ye, Shuai Kang, Youxiang Li, Shibin Sun, Ali Liu, Shuo Wang, Yuanli Zhao, Xiaolin Chen

**Affiliations:** 1 Department of Neurosurgery, Beijing Tiantan Hospital, Capital Medical University, Beijing, China; 2 China National Clinical Research Center for Neurological Diseases, Beijing, China; 3 Department of Neurosurgery, The First Hospital of Hebei Medical University, Hebei Medical University, Hebei, China; 4 Department of Interventional Neuroradiology, Beijing Tiantan Hospital, Capital Medical University, Beijing, China; 5 Gamma Knife Center, Beijing Tiantan Hospital, Capital Medical University, Beijing, China; 6 Department of Neurosurgery, Shanxi Provincial People’s Hospital, Shanxi, China; 7 Department of Neurosurgery, Peking University International Hospital, Peking University, Beijing, China; 8 Department of Neurosurgery, Affiliated Hospital of Xuzhou Medical University, Jiangsu, China; 9 Department of Neurosurgery, The First People’s Hospital of Qinzhou, Guangxi, China

## Abstract

**Importance:**

The dilemma between natural rupture risk and adverse outcomes of intervention is of major concern for patients with unruptured arteriovenous malformations (AVMs). The existing risk score for AVM rupture includes factors that are controversial and lacks prospective validation.

**Objective:**

To develop and robustly validate a reliable scoring system to predict the rupture risk of AVMs.

**Design, Setting, and Participants:**

This prognostic study developed a prediction model derived from a single-center cohort (derivation cohort) and validated in a multicenter external cohort (multicenter external validation cohort) and a cohort of patients receiving conservative treatment management (conservative treatment validation cohort). Patients were recruited from a nationwide multicenter prospective collaboration registry in China. A total of 4135 patients were enrolled in the registry between August 1, 2011, and September 1, 2021. Of those, 3962 patients were included in the study (3585 in the derivation cohort and 377 in the multicenter external validation cohort); 1028 patients from the derivation cohort who had time-to-event data and prerupture imaging results were included in the conservative treatment validation cohort. Data were analyzed from March 10 to June 21, 2022.

**Main Outcomes and Measures:**

A scoring system was developed based on risk factors identified from a literature review and a robust selection process. Patients were stratified into different risk groups based on scores to calculate hemorrhage-free probability in future years, and Kaplan-Meier curves were plotted to visualize risk stratification. Receiver operating characteristic curves were used to assess the discrimination of models. Univariable analyses (logistic regression analysis for descriptive data and Cox regression analysis for survival data) were used to compare baseline information and assess bias.

**Results:**

Among 3962 patients (2311 men [58.3%]; median [IQR] age, 26.1 [14.6-35.5] years), 3585 patients (2100 men [58.6%]; median [IQR] age, 25.9 [14.6-35.0] years) were included in the derivation cohort, and 377 patients (211 men [56.0%]; median [IQR] age, 26.4 [14.5-39.2] years) were included in the multicenter external validation cohort. Thirty-six hemorrhages occurred over a median (IQR) follow-up of 4.2 (0.3-6.0) years among 1028 patients in the conservative treatment validation cohort. Four risk factors were used to develop the scoring system: ventricular system involvement, venous aneurysm, deep location, and exclusively deep drainage (VALE). The VALE scoring system performed well in all 3 cohorts, with areas under the receiver operating characteristic curve of 0.77 (95% CI, 0.75-0.78) in the derivation cohort, 0.85 (95% CI, 0.81-0.89) in the multicenter external validation cohort, and 0.73 (95% CI, 0.65-0.81) in the conservative treatment validation cohort. The 10-year hemorrhage-free rate was 95.5% (95% CI, 87.1%-100%) in the low-risk group, 92.8% (95% CI, 88.8%-97.0%) in the moderate-risk group, and 75.8% (95% CI, 65.1%-88.3%) in the high-risk group; the model discrimination was significant when comparing these rates between the high-risk group and the low- and moderate-risk groups (*P* < .001 for both comparisons).

**Conclusions and Relevance:**

In this prognostic study, the VALE scoring system was developed to distinguish rupture risk among patients with AVMs. The stratification of unruptured AVMs may enable patients with low risk of rupture to avoid unnecessary interventions. These findings suggest that the scoring system is a reliable and applicable tool that can be used to facilitate patient and physician decision-making and reduce unnecessary interventions or unexpected AVM ruptures.

## Key Points

**Question:**

Can imaging data be used to derive a scoring system to estimate long-term risk of hemorrhage of unruptured brain arteriovenous malformations (AVMs)?

**Findings:**

In this prognostic study of 3962 patients with AVMs, 4 factors (ventricular system involvement, venous aneurysm, deep location, and exclusively deep drainage) were identified to build a scoring system to predict the rupture risk of AVMs. The scoring system had excellent performance in both the derivation and multicenter external validation cohorts and high discrimination in risk stratification of patients in the prospective follow-up cohort receiving conservative treatment management.

**Meaning:**

These findings suggest that the scoring system is a reliable tool that can be used to reduce unnecessary interventions or unexpected AVM ruptures.

## Teaser

This prognostic study uses data from a nationwide multicenter prospective registry in China to develop a scoring system that predicts the risk of hemorrhage among patients with brain arteriovenous malformations and validates the score in external multicenter and clinical practice cohorts.

## Introduction

Brain arteriovenous malformations (AVMs) are tangles of abnormally dilated vessels without intervening capillaries, which represent high-flow and low-resistance hemodynamic features due to direct arteriovenous shunting.^1,2^ Brain AVMs can lead to intracranial hemorrhage, with an annual rupture risk of 1% to 3% per year if left untreated.^3,4^ Although several grading scales have been proposed and are widely used to evaluate operability or treatment outcomes,^5,6,7,8^ it remains debatable whether interventional therapy can benefit patients with AVMs.^9,10,11,12^ Therefore, these options should be weighed against the patient’s natural hemorrhagic risks, which may vary widely across AVMs with different features.^13^

The R_2_eD score developed by Feghali et al^14^ was the first scale to estimate the rupture risk of AVMs and was externally validated by Bird et al.^15^ This scoring system identified 5 risk factors from statistical models (race [non-White], exclusive deep location, small AVM size, exclusive deep drainage, and monoarterial feeding). However, this scale should be applied and interpreted with caution due to its cross-sectional data–based selection of risk factors and its lack of prospective validation. Thus, clinicians and patients need a more reliable prediction and risk stratification tool for AVM rupture to inform expectations of hemorrhage-free outcomes and avoid unnecessary interventional treatment. Because the necessary imaging data were easily accessible for diagnosing AVMs, this study aimed to develop and validate a new scoring system combining evidence-based and statistically significant imaging features for the prediction of long-term hemorrhagic risk in patients with unruptured AVMs.

## Methods

### Study Design and Patients

In this prognostic study, we developed a prediction model for AVM rupture risk using prospectively collected data from a single-center cohort (derivation cohort) between August 1, 2011, and September 1, 2021. Data were analyzed from March 10 to June 21, 2022. Patients in this study were recruited from the MATCH (Multimodality Treatment for Brain Arteriovenous Malformation in Mainland China)^16^ nationwide multicenter prospective collaboration registry. The institutional review board of Beijing Tiantan Hospital approved this study, and patients who participated in the MATCH registry provided written informed consent at hospital admission. This study followed the Transparent Reporting of a Multivariable Prediction Model for Individual Prognosis or Diagnosis (TRIPOD) reporting guideline for prognostic studies.^17^

**Figure 1.**
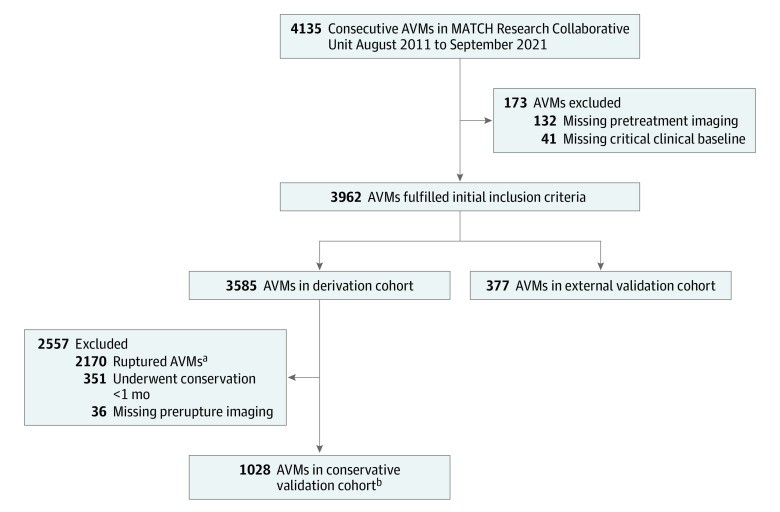
Flowchart of Patient Selection for Scoring System Development and Validation MATCH indicates Multimodality Treatment for Brain Arteriovenous Malformation in Mainland China. ^a^Patients were excluded if arteriovenous malformations (AVMs) were ruptured at admission unless they had received conservative treatment management more than 1 month after diagnosis. ^b^Among patients receiving conservative treatment management, 793 were retrospectively chosen for inclusion (ie, patients were diagnosed elsewhere and received interventional treatment in our institution more than 1 month after diagnosis), and 235 were prospectively followed up (ie, patients chose conservative treatment management after they were admitted to our institution).

Validation of this model consisted of 2 steps. A multicenter cohort involving patients from 8 provinces in mainland China was used to validate the model externally (multicenter external validation cohort). To further validate the generalizability of the model in a clinical practice scenario, we used data from patients with unruptured AVMs who were receiving conservative treatment management for more than 1 month (conservative treatment validation cohort). Only those with prerupture or pretreatment imaging data were eligible for inclusion in the study. The follow-up duration was censored at the date of hemorrhage or first treatment. The diagnosis of AVM was confirmed with digital subtraction angiography and/or magnetic resonance imaging (MRI). Those without essential imaging data for feature extraction and those diagnosed with dural AVMs, extracranial malformations, cavernous malformations, or hereditary hemorrhagic telangiectasia were excluded. The patient inclusion flowchart is shown in Figure 1.

### Data Collection and Variables of Interest

The comprehensive protocol for data quality management is available in eMethods 1 in Supplement 1. Demographic information, including sex, age, and modified Rankin Scale Score (which measures degree of disability or dependence after a stroke) at hospital admission, was recorded. Radiographic variables describing AVM morphological characteristics, including nidus location, size, diffuseness, venous drainage (drainage patterns, stenosis, and venous aneurysms), feeding arteries (number, dilation, multiple sources, and perforating arteries), associated aneurysm, and hemorrhagic presentation, were collected. Radiological information was determined via digital subtraction angiography and MRI.

The nidus location was regarded as deep if the lesion exclusively involved the brain stem, cerebellum, basal ganglia, thalamus, corpus callosum, or insular lobe. The definition of eloquent regions (ie, sensory, motor, language, or visual cortex; hypothalamus or thalamus; internal capsule; brain stem; cerebellar peduncles [superior, middle, or inferior]; and deep cerebellar nuclei) was based on the Spetzler-Martin Grading Scale.^5^ The size of AVMs was dichotomized into small and large based on whether the maximum nidal diameter was less than 3 cm or 3 cm or greater. Ventricular system involvement was determined via MRI based on whether the nidal border was adjacent to the cerebral ventricular system. Feeding arteries were considered dilated when their diameter was at least twice that of the same blood vessel segments. Venous aneurysm was defined as the focal aneurysmal dilation of the proximal drainage vein.^18^ Hemorrhagic presentation was defined as hemorrhage that could be ascribed to AVM rupture before or at admission.

### Model Performance and Validation

We used receiver operating characteristic curves (ROCs) to assess the discrimination of models in the derivation cohort and the 2 validation cohorts. An area under the receiver operating characteristic curve (AUROC) higher than 0.70 indicated a good prediction of rupture. The new model derived from the derivation cohort was evaluated by performing ROC analyses separately in these 3 cohorts. A calibration curve was plotted to investigate the calibration ability of the new model. A perfect prediction was defined as a line with a slope of 1 and an intercept of 0 in the calibration plot. Kaplan-Meier curves were plotted for the conservative treatment validation cohort to visualize risk stratification according to score. Survival analyses were conducted using the rms, version 6.3.0,^19^ and survival, version 3.4.0,^20^ packages for R software (R Foundation for Statistical Computing). Parameters in the R_2_eD score (except for race and ethnicity because the study population included only Chinese patients) were used to fit the model in our training set (data from the derivation cohort), and the DeLong test^14^ was conducted to compare the ROCs between the R_2_eD-based model and our model. Data from the 3 cohorts were also used for external validation of the R_2_eD score to investigate its generalizability beyond its majority White population.

### Statistical Analysis

We used R software, version 4.0.3 (R Foundation for Statistical Computing), to perform statistical data analyses. Statistical significance was set at 2-tailed *P* < .05. All data were divided into 2 groups according to rupture presentation. For continuous values, means with SDs or medians with IQRs were reported for normal and nonnormal distribution data. The proportion of each categorical variable was also recorded. We compared the baseline information of the derivation cohort between groups using univariable analysis, with a *t* test or Mann-Whitney *U* test used for continuous data and a χ^2^ test used for categorical data, as appropriate. Missing data were assumed to be missing at random. Missing values were imputed using the missForest package for R software, version 1.5.^21^ All available data from the derivation database were used to meet the 20 events per variable rule to maximize the power of our results.

We identified potential predictors based on background knowledge and findings from systematically reviewed literature that investigated these factors using a prospective follow-up design. Odds ratios (ORs) and hazard ratios (HRs) and 95% CIs for these variables were calculated. The most frequently mentioned variables in the published studies were considered central predictors and were fixed in models during the variable selection. We first fitted a global model with all variables, then used backward stepwise regression with the Akaike information criterion as the stopping criterion. We repeated the process in 1000 bootstrap resamples to investigate the stability of selected variables.^22^ The number of predictors was limited to 4 to develop a scoring scale that could be easily applied in clinical practice. The predictors in the final model were the combination of 2 clinically acknowledged factors and 2 statistically significant and stable factors. Multicollinearity was evaluated using the variance inflation factor (with >3 indicating high collinearity). We also used β coefficients to assign points for these predictors to establish a scoring system that could be conveniently used in clinical practice. We plotted the predicted probabilities of rupture risk using the new model (y-axis) and the total scores from the new grading system (x-axis). A description of the probability calculation is provided in eMethods 2 in Supplement 1.

## Results

### Patients and AVM Characteristics

A total of 4135 patients with AVMs were enrolled in the MATCH multicenter prospective registry. After careful screening, the final analysis included 3962 patients (2311 men [58.3%] and 1651 women [41.7%]; median [IQR] age, 26.1 [14.6-35.5] years); of those, 3585 patients (2100 men [58.6%] and 1485 women [41.4%]; median [IQR] age, 25.9 [14.6-35.0] years) from Beijing Tiantan Hospital (the MATCH registry sponsor) were in the derivation cohort, and 377 patients (211 men [56.0%] and 166 women [44.0%]; median [IQR] age, 26.4 [14.5-39.2] years) were in the multicenter external validation cohort. Hemorrhage occurred in 2189 patients (61.1%) in the derivation cohort and 225 patients (59.7%) in the multicenter external validation cohort. Missing values in the derivation cohort were less than 3% (eTable 1 in Supplement 1). Other baseline characteristics are shown in Table 1. The univariable analysis of the derivation cohort revealed that most of the investigated angioarchitectural features significantly differed between patients with AVM rupture (n = 2189) vs those without AVM rupture (n = 1396) (eTable 2 in Supplement 1). For example, AVMs with exclusively deep location and ventricular system involvement were associated with more hemorrhagic presentations (exclusively deep location: 693 patients [31.7%] with rupture vs 200 patients [14.3%] without rupture; *P* < .001; ventricular system involvement: 1477 patients [67.5%] with rupture vs 468 patients [33.5%] without rupture; *P* < .001). Feeding and draining patterns were also associated with AVM rupture (eg, single feeding artery: 889 patients [40.6%] patients with rupture vs 225 patients [16.1%] without rupture; *P* < .001; exclusively deep drainage: 721 patients [32.9%] with rupture vs 137 patients [9.8%] without rupture; *P* < .001).

**Table 1.** Cohort Characteristics

Characteristic	Patients, No./total No. (%)		
	Derivation cohort (n = 3585)^a^	Validation cohorts	
		Multicenter external (n = 377)	Conservative treatment management (n = 1028)
Sex			
Female	1485/3585 (41.4)	166/377 (44.0)	401/1028 (39.0)
Male	2100/3585 (58.6)	211/377 (56.0)	627/1028 (61.0)
Age at diagnosis, median (IQR), y	25.9 (14.6-35.0)	26.4 (14.5-39.2)	27.2 (17.1-37.5)
Hemorrhagic presentation	2189/3585 (61.1)	225/377 (59.7)	36/1028 (3.5)
Seizure	853/3585 (23.8)	81/377 (21.5)	415/1028 (40.4)
AVM features			
Size, cm			
<3	1778/3585 (49.6)	201/377 (53.3)	343/1028 (33.4)
3-6	1456/3585 (40.6)	157/377 (41.6)	517/1028 (50.3)
>6	351/3585 (9.8)	19/377 (5.0)	168/1028 (16.3)
Nidus location			
Frontal lobe	912/3585 (25.4)	55/377 (14.6)	341/1028 (33.2)
Temporal lobe	981/3585 (27.4)	85/377 (22.5)	286/1028 (27.8)
Parietal lobe	944/3585 (26.3)	84/377 (22.3)	306/1028 (29.8)
Occipital lobe	733/3585 (20.4)	57/377 (15.1)	226/1028 (22.0)
Cerebellum	339/3585 (9.5)	58/377 (15.4)	61/1028 (5.9)
Brain stem	117/3585 (3.3)	17/377 (4.5)	24/1028 (2.3)
Basal ganglia	390/3585 (10.9)	69/377 (18.3)	55/1028 (5.4)
Thalamus	208/3585 (5.8)	34/377 (9.0)	35/1028 (3.4)
Intraventricular area	154/3585 (4.3)	27/377 (7.2)	29/1028 (2.8)
Insula	72/3585 (2.0)	26/377 (6.9)	18/1028 (1.8)
Exclusively deep location	893/3585 (24.9)	131/377 (34.7)	143/1028 (13.9)
Ventricular system involvement	1945/3585 (54.3)	187/377 (49.6)	330/1028 (32.1)
Eloquent region	2000/3585 (55.8)	252/377 (66.8)	535/1028 (52.0)
Feeding artery			
Single feeder	1081/3491 (31.0)	120/377 (31.8)	179/1028 (17.4)
Dilation	1641/3518 (46.6)	224/377 (59.4)	685/1028 (66.6)
Multiple sources	976/3525 (27.7)	98/377 (26.0)	375/1028 (36.5)
Perforating artery	1333/3519 (37.9)	157/377 (41.6)	308/1028 (30.0)
Diffuse nidus	1261/3508 (35.9)	124/377 (32.9)	222/1028 (21.6)
Venous draining			
Stenosis	551/3527 (15.6)	89/377 (23.6)	108/1028 (10.5)
Any deep drainage	1415/3528 (40.1)	170/377 (45.1)	305/1028 (29.7)
Exclusively deep drainage	850/3528 (24.1)	107/377 (28.4)	92/1028 (8.9)
Venous aneurysm	565/3519 (16.1)	97/377 (25.7)	320/1028 (31.1)
Aneurysm	592/3510 (16.9)	80/377 (21.2)	157/1028 (15.3)
Spetzler-Martin grade^b^			
I	562/3528 (15.9)	56/377 (14.9)	157/1028 (15.3)
II	1180/3528 (33.4)	114/377 (30.2)	312/1028 (30.4)
III	1162/3528 (32.9)	126/377 (33.4)	352/1028 (34.2)
IV	494/3528 (14.0)	73/377 (19.4)	151/1028 (14.7)
V	130/3528 (3.7)	8/377 (2.1)	56/1028 (5.4)
Spetzler-Martin combined grade^c^			
II	81/3495 (2.3)	5/377 (1.3)	1/1028 (0.1)
III	306/3495 (8.8)	30/377 (8.0)	37/1028 (3.6)
IV	705/3495 (20.2)	66/377 (17.5)	119/1028 (11.6)
V	946/3495 (27.1)	106/377 (28.1)	250/1028 (24.3)
VI	807/3495 (23.1)	98/377 (26.0)	297/1028 (28.9)
VII	462/3495 (13.2)	55/377 (14.6)	207/1028 (20.1)
VIII	147/3495 (4.2)	14/377 (3.7)	93/1028 (9.0)
IX	37/3495 (1.1)	2/377 (0.5)	24/1028 (2.3)
X	4/3495 (0.1)	1/377 (0.3)	0

Abbreviation: AVM, arteriovenous malformation.

^a^ The breakdown of missing values per parameter in the derivation cohort is provided in eTable 1 in Supplement 1.

^b^ Range, I-V, with higher grades indicating greater risk of surgical morbidity and death.

^c^ Spetzler-Martin grade combined with supplemental Spetzler-Martin grade (which includes only statistically significant variables not already included in the original Spetzler-Martin Grading Scale); range, II-X, with higher grades indicating greater risk of surgical morbidity and death.

A total of 1028 patients from the derivation cohort who had time-to-event data and prerupture imaging results were included in the conservative treatment validation cohort. Among those, 793 patients receiving conservative treatment management (77.1%) were retrospectively chosen for inclusion (retrospective group, comprising patients who were diagnosed elsewhere and received interventional treatment in our institution more than 1 month after diagnosis), and 235 patients were prospectively followed up (prospective follow-up group, comprising patients who chose to receive conservative treatment management after admission to our institution). Thirty-six hemorrhages occurred in the conservative treatment validation cohort (22.9%), with a median (IQR) follow-up duration of 4.2 (0.3-6.0) years. The annual hemorrhage rate for the total cohort was 0.84% (0.81% for the retrospective group and 0.87% for the prospective follow-up group). The overall and subgroup Kaplan-Meier curves are shown in eFigure 1 in Supplement 1. No significant differences were observed.

### Development of the New Scoring System

Based on the results of the literature review, we summarized factors associated with AVM rupture that were reported in studies with prospective designs^13,23,24,25,26,27,28,29,30,31,32,33,34,35^ (eTable 3 in Supplement 1). In addition to previous hemorrhagic history, deep location and deep drainage were the most frequently mentioned factors. These 2 variables were therefore deemed central to our score. Backward stepwise regression analysis was used to select 10 of 12 variables with corresponding coefficients, and a bootstrapping process was used to investigate the stability of these factors. A summary of the findings from this analysis is provided in eTable 4 in Supplement 1. Because we considered 4 variables to be the most appropriate number to include in a practical prediction model, only the top 2 factors (ventricular system involvement and venous aneurysm) were added to the final model along with deep location and exclusively deep drainage. The univariable analyses (logistic regression analysis for descriptive data and Cox regression analysis for survival data) in the 3 study cohorts revealed that the effect sizes of the factors could have heterogeneity in different analyses. Univariable analyses (logistic regression analysis for descriptive data and Cox regression analysis for survival data) revealed that ventricular system involvement (OR, 4.11 [95% CI, 3.57-4.74] for the derivation cohort; OR, 8.82 [95% CI, 5.43-14.34] for the multicenter external validation cohort; and HR, 4.04 [95% CI, 2.02-8.08] for the conservative treatment validation cohort), venous aneurysm (OR, 0.15 [95% CI, 0.13-0.19] for the derivation cohort; OR, 0.09 [95% CI, 0.05-0.15] for the multicenter external validation cohort; and HR, 0.42 [95% CI, 0.17-1.00] for the conservative treatment validation cohort), deep location (OR, 2.77 [95% CI, 2.33-3.30] for the derivation cohort; OR, 2.63 [95% CI, 1.65-4.18] for the multicenter external validation cohort; and HR, 4.39 [95% CI, 2.22-8.70] for the conservative treatment validation cohort), and exclusively deep drainage (OR, 4.51 [95% CI, 3.70-5.50] for the derivation cohort; OR, 6.32 [95% CI, 3.48-11.46] for the multicenter external validation cohort; and HR, 3.60 [95% CI, 1.63-7.96] for the conservative treatment validation cohort) were significant in all 3 cohorts (eTable 6 in Supplement 1).

Information on the newly developed scoring system (VALE, an acronym based on the 4 included risk factors: ventricular system involvement, venous aneurysm, deep location, and exclusively deep drainage), with corresponding estimated β coefficients and SEs from the final regression model, is shown in Table 2. One point was assigned to AVMs with deep location (OR, 1.45; 95% CI, 1.17-1.78), and 2 points each were assigned to AVMs with ventricular system involvement (OR, 3.27; 95% CI, 2.79-3.83) and exclusively deep drainage (OR, 2.30; 95% CI, 1.83-2.90). Venous aneurysm was a protective factor for AVM rupture in the study cohort (OR, 0.16; 95% CI, 0.13-0.20); therefore, −4 points were assigned if venous aneurysm presented. An analysis of variance inflation factors revealed no significant collinearity between these variables. The total VALE score ranged from −4 to 5 points. As shown in eFigure 2 in Supplement 1, the predicted probability of rupture increased as the VALE score increased. According to the hemorrhagic probability, we further categorized the VALE score into 3 groups for clinical practicality: low risk (score of less than −2), moderate risk (score of −2 to 1), and high risk (score of greater than 1).

**Table 2.** VALE Score and Corresponding Estimated Coefficients and SEs in the Final Model

Acronym	Predictor	β Coefficient (SE)	Points assigned^a^
V	Ventricular system involvement	1.185 (0.081)	2
A	Venous aneurysm	−1.854 (0.112)	−4
L	Deep location	0.371 (0.107)	1
E	Exclusively deep drainage	0.833 (0.117)	2

Abbreviation: VALE, ventricular system involvement, venous aneurysm, deep location, and exclusively deep drainage.

^a^ The points were originally determined by rounding the logistic regression model coefficients (1 for V, −2 for A, 0.5 for L, and 1 for E). To develop a scoring system with integral values, these points were doubled to form the score on a scale of 10.

### Model Performance and Validation

**Figure 2.**
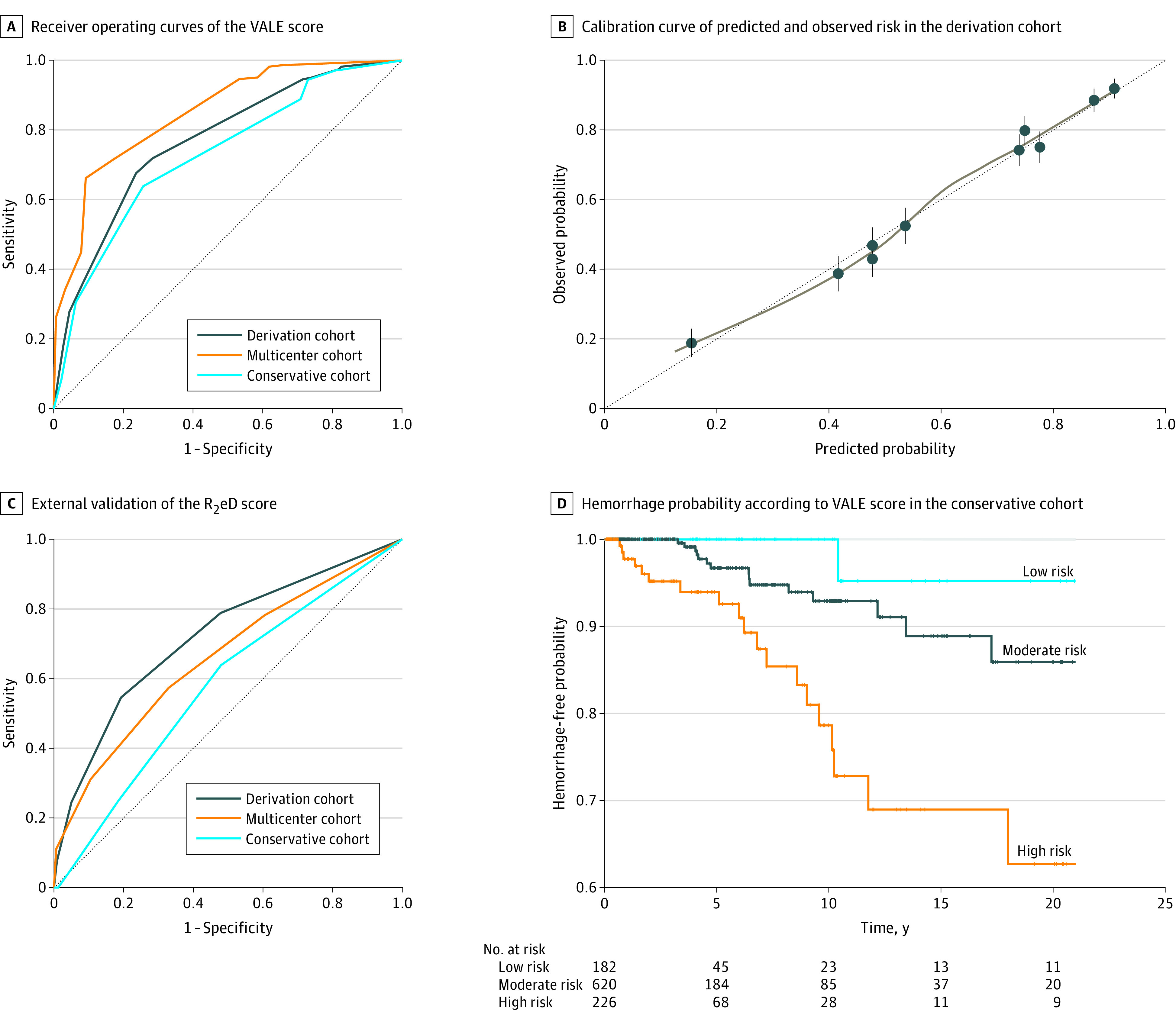
Performance of the VALE Scoring System and Comparison With the R_2_eD Scoring System In panel C, the area under the receiver operating characteristic curve was 0.579. In panel D, *P* values were .12 for low risk vs moderate risk, <.001 for low risk vs high risk, and <.001 for moderate risk vs high risk. No hemorrhagic events occurred after 20 years of follow-up; therefore, the survival plot was cut off at 20 years for better data presentation. R_2_eD indicates race (non-White), exclusive deep location, small arteriovenous malformation, exclusive deep drainage, and monoarterial feeding, and monoarterial feeding (lesion with a single feeding artery); and VALE, ventricular system involvement, venous aneurysm, deep location, and exclusively deep drainage.

The performance of the VALE score for predicting AVM rupture in the 3 data sets is shown in Figure 2A. The AUROCs were 0.77 (95% CI, 0.75-0.78) in the derivation cohort, 0.85 (95% CI, 0.81-0.89) in the multicenter external validation cohort, and 0.73 (95% CI, 0.65-0.81) in the conservative treatment validation cohort. The calibration curve (Figure 2B) suggested good agreement between observation and prediction. We also performed a subgroup analysis of the conservative treatment validation cohort based on whether patients were in the prospective follow-up group or the retrospective group. Similar high discrimination was observed for both the prospective follow-up group (AUROC, 0.75; specificity, 0.74; sensitivity, 0.61) and the retrospective group (AUROC, 0.75; specificity, 0.77; sensitivity, 0.63) (eTable 5 in Supplement 1). A risk stratification analysis was also conducted for the conservative treatment validation data set, and the Kaplan-Meier curves are shown in Figure 2D. The groups with low and moderate risk shared similar rupture risk in the long-term follow-up (eg, 10-year hemorrhage-free probability: 95.5% [95% CI, 87.1%-100%] for low-risk group vs 92.8% [95% CI, 88.8%-97.0%] for moderate-risk group; *P* = .12), while the differences between these 2 groups and the high-risk group were significant (eg, 10-year hemorrhage-free probability for high-risk group: 75.8% [95% CI, 65.1%-88.3%]; *P* < .001 for comparisons with both low- and moderate-risk groups). Hemorrhage-free probability among patients in the conservative treatment validation cohort at 3 years, 5 years, and 10 years is shown in Table 3.

**Table 3.** Hemorrhage-Free Probability Stratified by Risk Group in the Conservative Treatment Validation Cohort

Risk group^a^	Hemorrhage-free probability by years of follow-up, % (95% CI)		
	3	5	10
Low	100 (ND)	100 (ND)	95.5 (87.1-100)
Moderate	99.6 (98.8-100)	96.7 (94.3-99.1)	92.8 (88.8-97.0)
High	95.2 (91.5-99.0)	92.6 (87.6-97.8)	75.8 (65.1-88.3)

Abbreviation: ND, not defined.

^a^ Low risk was defined as a VALE score of less than −2, moderate risk as a VALE score of −2 to 1, and high risk as a VALE score of greater than 1.

We also investigated the performance of a previously published risk score for AVM rupture (the R_2_eD score) as the largest external validation set for our VALE score.^14^ The R_2_eD-based model fitted to the derivation cohort of the present study had good discrimination (AUROC, 0.73; 95% CI, 0.71-0.74), but the VALE model performed better (AUROC, 0.77; 95% CI, 0.76-0.79; DeLong test *P* < .001). The ROC of the R_2_eD score fitted to the 3 study cohorts, plotted separately, is shown in Figure 2C. The R_2_eD score performed well in the derivation and multicenter external validation cohorts, with AUROCs of 0.72 (95% CI, 0.70-0.74) and 0.66 (95% CI, 0.61-0.71), respectively. However, in the conservative treatment validation cohort with survival data, the discrimination of the R_2_eD score was worse than that of the R_2_eD score in the other 2 cohorts (AUROC, 0.58; 95% CI, 0.49-0.67). The VALE score better predicted AVM rupture in all cohorts, especially in the cohort simulating a clinical practice scenario (ie, the conservative treatment validation cohort). A description of how to use the VALE score in clinical practice, with detailed examples, is available in eFigure 3 in Supplement 1.

## Discussion

This prognostic study identified 4 risk factors associated with AVM rupture (ventricular system involvement, venous aneurysm, deep location, and exclusively deep drainage) and established the first prospectively validated scoring system (the VALE score). The VALE score had good performance in the derivation cohort and was well validated in the multicenter external cohort and the conservative treatment cohort, with AUROCs of 0.85 and 0.73, respectively. Long-term follow-up of the conservative treatment validation cohort enabled the VALE scoring system to predict survival over different periods across risk groups, facilitating decision-making for both clinicians and patients.

The R_2_eD scoring system includes 5 risk factors identified from statistical models (non-White race, exclusive deep location, small AVM sizes, exclusive deep drainage, and monoarterial feeding).^14^ The VALE score shares similar purposes and 2 variables with the R_2_eD score: deep location and exclusively deep drainage. In our model, we selected these 2 acknowledged risk factors based on literature review,^13,23,24,25,26,27,28,29,30,31,32,33,34,35^ with the aim of avoiding results that may have been misleading because they were statistically significant but lacked clinical relevance. This issue is a limitation of other variables included in the R_2_eD score; for example, the risk associated with race may instead (or also) be associated with socioeconomic status,^14^ and the inclusion of small AVM size in risk scores has long been debated, with the prevailing view in opposition to its inclusion.^23^ The discrepancies caused by selection bias are inevitable when using cross-sectional data. We conducted univariable analyses (logistic regression analysis for descriptive data and Cox regression analysis for survival data) in the 3 study cohorts to further investigate biases. Deep location and exclusively deep drainage remained significant in all 3 cohorts, which was consistent with the findings of previous studies.^13,24,25,26,27,28,36^ Among other predictors included in the VALE score, ventricular system involvement also had high predictive power in all groups, and venous aneurysm was nonsignificant in the prospective follow-up group within the conservative treatment validation cohort. The heterogeneity of the effect size suggested that selecting variables simply by using descriptive data analysis was unstable and unreliable. A study assessing the performance of AVM rupture prediction models in terms of methods used^37^ also found unstable results in models derived from data sets with different sample sizes and sampling times.

A better variable selection process can be derived by using prospective cohorts with time-to-event data. However, an international multicenter prospective study,^29^ which was limited by small sample size and low incidence of rupture, did not identify any angioarchitectural features. Therefore, the present study conducted a standardized variable selection procedure based on bootstrap resampling,^22^ combining evidence-based risk factors with statistically significant predictors to develop an easily applicable score that was then validated in a conservative treatment cohort with survival data. The good performance in the validation cohort enhanced the scoring system’s applicability in a clinical practice scenario.

When considering potential mechanisms of risk for the angioarchitectural features included in the VALE score, ventricular system involvement may lead to abnormal transmural pressure that makes it difficult to maintain the structural stability of the vessel wall due to lack of support from brain tissue. Venous aneurysms have been found to have a highly protective benefit for rupture despite the inconsistency of findings across studies.^26,38^ One possible reason may be that the dilated cavity could serve as a buffer zone to reduce impedance of the outflow.

Results from the Randomized Trial of Unruptured Brain Arteriovenous Malformations^9^ suggested that conservative treatment management was superior to intervention for the prevention of stroke. This conclusion has been controversial because of study design aspects.^12,39^ Treatment of AVMs is complicated, and individual differences in clinical decision-making should be considered. Many scoring scales have been developed in the AVM field, and they should be applied to different elements of the treatment process. In our study, the VALE score was developed for risk stratification of patients with different features of unruptured AVMs. Patients in different rupture risk groups are typically recommended to receive individualized therapeutic strategies based on assessment by different scales: the Spetzler-Martin and supplementary Spetzler-Martin grades for surgical operability,^5,6^ the Buffalo and Arteriovenous Malformation Embocure scores for endovascular treatment,^8,40^ and the Virginia Radiosurgery AVM Scale for radiosurgical procedures.^7^ For patients in different risk groups, the natural history of AVM rupture and the benefit of different treatment strategies must be weighed separately. Multicenter prospective validation with long-term follow-up using these scoring scales comprehensively is also required to better guide clinical practice.

### Limitations

This study has several limitations. First, to serve as an applicable tool in clinical practice, our model only included 4 variables derived from imaging data despite the identification of other significant factors in the selection process and literature review.^29,41,42^ This restriction to 4 risk factors may, to some extent, reduce the accuracy of the score while increasing its convenience. With deeper investigation of genetic information and radiomic data, a larger number of variables associated with AVM rupture could be uncovered, at which point artificial intelligence could be used to develop a more comprehensive and precise model. Second, the relatively low number of hemorrhagic events in the conservative treatment validation cohort produced a lower annual rupture rate than that of other studies.^23,28,29^ This difference can partly be ascribed to the exclusion of patients with ruptured AVMs but without prerupture imaging data in the retrospective group within the conservative treatment validation cohort. Selection bias can result in variations in incidence rates but does not change the association between these factors and rupture risk. That said, the hemorrhage-free survival probability derived from this cohort should be interpreted with caution because it might be overestimated. Third, development and validation of the VALE scoring system were performed in a predominantly Chinese population, so its generalizability to other populations remains to be assessed by other international collaborations, such as the Multicenter AVM Research Study.^29^

## Conclusions

In this prognostic study, the VALE scoring system, consisting of 4 central imaging features, was developed to stratify the risk of rupture among patients with AVM and provide estimated hemorrhage-free probability for future years. The stratification of unruptured AVMs may enable patients with low risk of rupture to avoid unnecessary interventions. These findings suggest the VALE scoring system is a reliable and applicable tool that may be used to help identify those who can benefit from early intervention, reduce unnecessary interventions or unexpected AVM ruptures, facilitate decision-making for both clinicians and patients, and guide future clinical research toward more personalized design.

## Notes

Supplement 1. **eMethods 1.** Protocol for Data Quality Management **eMethods 2.** Calculation of Predicted Hemorrhage Probability According to VALE Score **eTable 1.** Breakdown of Missing Data in the Derivation Cohort **eTable 2.** Univariable Analysis of the Derivation Cohort **eFigure 1.** Kaplan-Meier Curves of Overall and Subgroup Hemorrhage-Free Probability in the Conservative Treatment Validation Cohort **eTable 3.** Literature Review of Factors Associated With Arteriovenous Malformation Rupture **eTable 4.** Variable Selection and Bootstrap-Derived Quantities Useful for Assessing Stability **eFigure 2.** Hemorrhage Probability in the Derivation Cohort **eTable 5.** Subgroup Analysis of VALE Score in the Conservative Treatment Validation Cohort According to the Retrospective and Prospective Nature of the Survival Data **eFigure 3.** Illustration Cases for the VALE Score **eTable 6.** Univariable Analysis of Angiographic Features in Different Cohorts
eReferences

Supplement 2.
Data Sharing Statement
