# Supplementary material for: Development and Validation of a Scoring System for Hemorrhage Risk in Brain Arteriovenous Malformations
Source: JAMA Netw Open. 2023 Mar 1;6(3):e231070. doi: 10.1001/jamanetworkopen.2023.1070 (PMC9978947; doi:10.1001/jamanetworkopen.2023.1070)
Supplement: Supplement 1. — eMethods 1. Protocol for Data Quality Management eMethods 2. Calculation of Predicted Hemorrhage Probability According to VALE Score eTable 1. Breakdown of Missing Data in the Derivation Cohort eTable 2. Univariable Analysis of the Derivation Cohort eFigure 1. Kaplan-Meier Curves of Overall and Subgroup Hemorrhage-Free Probability in the Conservative Treatment Validation Cohort eTable 3. Literature Review of Factors Associated With Arteriovenous Malformation Rupture eTable 4. Variable Selection and Bootstrap-Derived Quantities Useful for Assessing Stability eFigure 2. Hemorrhage Probability in the Derivation Cohort eTable 5. Subgroup Analysis of VALE Score in the Conservative Treatment Validation Cohort According to the Retrospective and Prospective Nature of the Survival Data eFigure 3. Illustration Cases for the VALE Score eTable 6. Univariable Analysis of Angiographic Features in Different Cohorts eReferences [file jamanetwopen-e231070-s001.pdf]

Supplementary Online Content

Chen Y, Han H, Meng X, et al. Development and validation of a scoring system for hemorrhage risk in brain arteriovenous malformations. *JAMA Netw Open*. 2023;6(3):e231070. doi:10.1001/jamanetworkopen.2023.1070

eMethods 1. Protocol for Data Quality Management

eMethods 2. Calculation of Predicted Hemorrhage Probability According to VALE Score

eTable 1. Breakdown of Missing Data in the Derivation Cohort

eTable 2. Univariable Analysis of the Derivation Cohort

eFigure 1. Kaplan-Meier Curves of Overall and Subgroup Hemorrhage-Free Probability in the Conservative Treatment Validation Cohort

eTable 3. Literature Review of Factors Associated With Arteriovenous Malformation Rupture

eTable 4. Variable Selection and Bootstrap-Derived Quantities Useful for Assessing Stability

eFigure 2. Hemorrhage Probability in the Derivation Cohort

eTable 5. Subgroup Analysis of VALE Score in the Conservative Treatment Validation Cohort According to the Retrospective and Prospective Nature of the Survival Data

eFigure 3. Illustration Cases for the VALE Score

eTable 6. Univariable Analysis of Angiographic Features in Different Cohorts

eReferences

This supplemental material has been provided by the authors to give readers additional information about their work.

© 2023 Chen Y et al. *JAMA Network Open*.

eMethods 1. Protocol for Data Quality Management

1. Definition of variables were discussed and unified according to the terminology reporting standards or published paper before the initiation of data collection. Clinical research coordinators (CRCs) and neurosurgery residents were then trained by a cerebrovascular neurosurgeon with more than 15 years' working experience. CRCs were responsible for demographic information and follow-up data, and neurosurgery residents for angiographic features. The two parts were blinded to each other to ensure the data collected were not biased by imaging characteristics or clinical outcomes.
2. A standard training dataset with 50 cases were used to check the consistency of data collectors. For those variables or cases with significant interobserver variation, the consensus was reached by either modifying the confusing definitions or retraining the data collectors. Only when the consistency reached 90% can the CRC or the resident allowed to extracting information independently.
3. While recording data, one could ask for help about unsure cases in a discussion group with cerebrovascular neurosurgeons in it, or mark these cases and discuss in weekly meetings.
4. The group leader with more than five years' working experience randomly spot checks these data biweekly. Investigators would receive training again if their data were of low quality, and these data would be recollected by other investigators.

© 2023 Chen Y et al. *JAMA Network Open*.

eMethods 2. Calculation of Predicted Hemorrhage Probability According to VALE Score
eTable 1. Breakdown of Missing Data in the Derivation Cohort

The finally selected variables would be included in a logistic regression model with the binomial outcome of rupture presentation.

1. Final predictors with β-coefficients and the intercept would form a linear regression equation. In this study, the equation was as follows:

$$y = -0.090 + 1.185\ (Ventricular\ system\ involvement) - 1.854\ (Venous\ aneurym) + 0.371\ (Deep\ location) + 0.833\ (Exclusively\ deep\ drainage)$$

2. Plugging the results into the sigmoid function formula could generate a number between 0 to 1. The value represents the predicted hemorrhage probability of each individual.

$$Predicted\ value = \frac{e^{y}}{1 + e^{y}}$$

© 2023 Chen Y et al. *JAMA Network Open*.

eTable 1. Breakdown of Missing Data in the Derivation Cohort

Variable	Derivation Cohort, No. (%) (n = 3585)
Female	-
Age at diagnosis, y, median (IQR)	-
Hemorrhagic presentation	-
Seizure	-
Size (cm)	-
Location	-
Exclusively deep location	-
Ventricular system involvement	-
Eloquent region	-
Feeding artery	-
Single feeder	94 (2.6)
Dilation	67 (1.9)
Multiple source	60 (1.7)
Perforating artery	66 (1.8)
Diffuse nidus	77 (2.1)
Draining vein	-
Stenosis	58 (1.6)
Any deep drainage	57 (1.6)
Exclusive deep drainage	57 (1.6)
Venous aneurysm	66 (1.8)
Aneurysm	75 (2.1)
Spetzler-Martin grade	57 (1.6)
Supplemented Spetzler-Martin grade	90 (2.5)

Abbreviation: IQR, interquartile range.

© 2023 Chen Y et al. *JAMA Network Open*.

eTable 2. Univariable Analysis of the Derivation Cohort

Characteristic	No. (%)		P
	Ruptured AVM (n = 2189)	Unruptured AVM (n = 1396)	
Female	950 (43.4)	535 (38.3)	0.003
Age at diagnosis, y, median (IQR)	22.2 (13.0 – 32.9)	26.8 (17.0 – 37.5)	<0.001
Seizure	270 (12.3)	583 (41.8)	<0.001
Size (cm)			<0.001
<3	1321 (60.3)	457 (32.7)	
3-6	746 (34.1)	710 (50.9)	
>6	122 (5.6)	229 (16.4)	
Location			<0.001
Frontal	456 (20.8)	456 (32.7)	<0.001
Temporal	591 (27.0)	390 (27.9)	0.565
Parietal	521 (23.8)	423 (30.3)	<0.001
Occipital	423 (19.3)	310 (22.2)	0.041
Cerebellum	249 (11.4)	90 (6.4)	<0.001
Brain stem	81 (3.7)	36 (2.6)	0.081
Basal ganglia	313 (14.3)	77 (5.5)	<0.001
Thalamus	166 (7.6)	40 (3.0)	<0.001
Intra-ventricle	111 (5.1)	43 (3.1)	0.005
Insula	49 (2.2)	23 (1.6)	0.268
Exclusively deep location	693 (31.7)	200 (14.3)	<0.001
Infratentorial location	301 (13.8)	99 (7.1)	<0.001
Ventricular system involvement	1477 (67.5)	468 (33.5)	<0.001
Eloquent region	1281 (58.5)	719 (51.5)	<0.001
Feeding artery			
Single feeder	889 (40.6)	225 (16.1)	<0.001
Dilation	755 (34.5)	909 (65.1)	<0.001
Multiple source	468 (21.4)	523 (37.5)	<0.001
ACA supply	555 (25.4)	496 (36.8)	<0.001
MCA supply	1260 (59.2)	935 (69.4)	<0.001
PCirA supply	859 (39.2)	523 (37.5)	0.303
Perforating artery	928 (42.4)	419 (30.0)	<0.001
Diffuse nidus	995 (45.5)	320 (22.9)	<0.001
Draining vein			
Stenosis	413 (18.9)	152 (10.9)	<0.001
Deep drainage	1009 (46.1)	415 (29.7)	<0.001
Exclusive deep drainage	721 (32.9)	137 (9.8)	<0.001
Venous aneurysm	141 (6.4)	431 (30.9)	<0.001
Aneurysm	402 (18.4)	197 (14.1)	0.001

© 2023 Chen Y et al. *JAMA Network Open*.

Spetzler-Martin grade			<0.001
1	371 (16.9)	201 (14.4)	
2	751 (34.3)	453 (32.4)	
3	721 (32.9)	458 (32.8)	
4	297 (13.6)	203 (14.5)	
5	49 (2.2)	81 (5.8)	
Supplemented Spetzler-Martin grade			<0.001
2	82 (3.7)	0 (0.0)	
3	275 (12.6)	44 (3.2)	
4	562 (25.7)	162 (11.6)	
5	631 (28.8)	337 (24.1)	
6	435 (19.9)	396 (28.4)	
7	175 (8.0)	297 (21.3)	
8	25 (1.1)	123 (8.8)	
9	4 (0.2)	33 (2.4)	
10	0 (0.0)	4 (0.3)	

Abbreviation: IQR, interquartile range.

© 2023 Chen Y et al. *JAMA Network Open*.

eFigure 1. Kaplan-Meier Curves of Overall and Subgroup Hemorrhage-Free Probability in the Conservative Treatment Validation Cohort

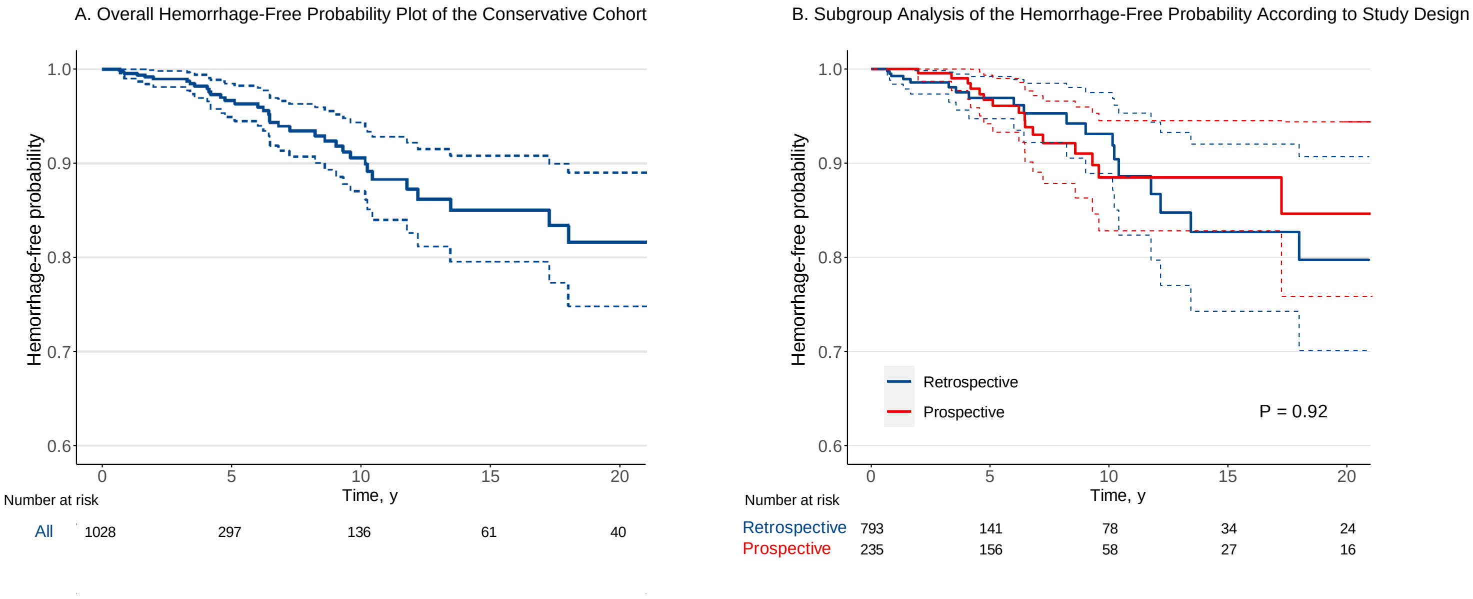

As no more hemorrhage event occurred after the 20 years' follow-up and the survival plot was then cut off there for better presentation of the data.

© 2023 Chen Y et al. *JAMA Network Open*.

eTable 3. Literature Review of Factors Associated With Arteriovenous Malformation Rupture

Risk Factor	Studies
Hemorrhage history	P M Crawford, 1986[1]; H Mast, 1997[2]; C Stapf, 2006[3]; H Kim, 2007[4]; J A Hernesniemi, 2008[5]; L da Costa, 2009[6]
Deep location	Y Itoyama, 1989[7]; S Mine, 2000[8]; M A Stefani, 2002[9]; C Stapf, 2006[3]; S Yamada, 2007[10]
Deep drainage	H Mast, 1997[2]; C Stapf, 2006[3]; L da Costa, 2009[6]
Large nidus size	S Mine, 2000[8]; M A Stefani, 2002[9]; J A Hernesniemi, 2008[5]
Small nidus size	C J Graf, 1983[11]; Y Itoyama, 1989[7]; R F Spetzler, 1992[12]
Infratentorial location	S Mine, 2000[8]; J A Hernesniemi, 2008[5]
Increasing age	P M Crawford, 1986[1]; C Stapf, 2006[3]; H Kim, 2014[13]
Aneurysm	L da Costa, 2009[6]
Posterior fossa	D Fults, 1984[14]
Female	S Yamada, 2007[10]
Male	H Mast, 1997[2]

© 2023 Chen Y et al. *JAMA Network Open*.

eTable 4. Variable Selection and Bootstrap-Derived Quantities Useful for Assessing Stability

Predictors	Global model [a]		Bootstrap inclusion frequency [b] (%)	Selected model [c]		RMSD ratio [d]	Bootstrap median [e]	Bootstrap 2.5th percentile	Bootstrap 97.5th percentile	Variance inflation factor [f]
	Estimate	SE		Estimate	SE					
Deep location (fixed)	−0.3584	0.1244	100	0.1389	0.1158	7.1225	0.1196	-0.1377	0.3806	1.2366
Exclusively deep drainage (fixed)	1.5335	0.1327	100	0.7088	0.1266	7.8840	0.7482	0.4770	1.0083	1.2591
Venous aneurysm	−1.5968	0.1190	100	−1.5697	0.1189	16.3479	−1.5768	−1.8106	−1.3334	1.0384
Ventricular system involvement	1.5335	0.0932	100	1.4057	0.0910	16.3920	1.4214	1.2339	1.604	1.1931
Venous stenosis	0.9139	0.1198	100	0.9364	0.1197	9.8174	0.9428	0.6968	1.2185	1.0246
Single feeder	0.8966	0.1074	100	0.8651	0.1068	10.4198	0.8717	0.6465	1.0753	1.2112
Large nidus size	−0.8445	0.0930	100	−0.7351	0.0924	13.5071	−0.7478	−0.9266	−0.5604	1.2133
Diffuse nidus	0.7959	0.0904	100	0.8127	0.0898	12.1001	0.8210	0.6477	1.0037	1.0049
Aneurysm	0.5154	0.1151	99.9	0.5166	0.1151	8.1827	0.5151	0.3093	0.7526	1.0231
Multiple source feeding	−0.3692	0.1010	97.3	−0.3452	0.1002	9.9326	−0.3407	−0.5508	0	1.2379
Eloquent region	0.1284	0.0912	33.9	0	0	9.5311	0	0	0.269	-
Perforating artery supply	0.1699	0.1039	31.2	0	0	8.5867	0	−0.3088	0	-

Abbreviations: SE, standard error; RMSD, root mean squared difference.

[a] The global model includes all candidate variables with regression coefficients and standard errors.

[b] Bootstrap inclusion frequencies are used to quantify how likely a variable is selected.

[c] Selected model is the model selected by Akaike information criterion using backward stepwise regression, and in our study, we didn't apply all these variables to the scoring system for the applicability in clinical practice. Variables were ranked and selected by the estimate for the final development of the scoring system.

[d] RMSD ratio is RMSD divided by the standard error of that coefficient in the global model, intuitively expresses the variance inflation or deflation caused by variable selection.

[e] Bootstrap median and the 95% CI are the coefficients estimated in the bootstrap procedure.

[f] The variance inflation factor was calculated to assess the multicollinearity of the finally significant 10 variables. The results showed no significant collinearity was observed across these variables.

© 2023 Chen Y et al. *JAMA Network Open*.

eFigure 2. Hemorrhage Probability in the Derivation Cohort

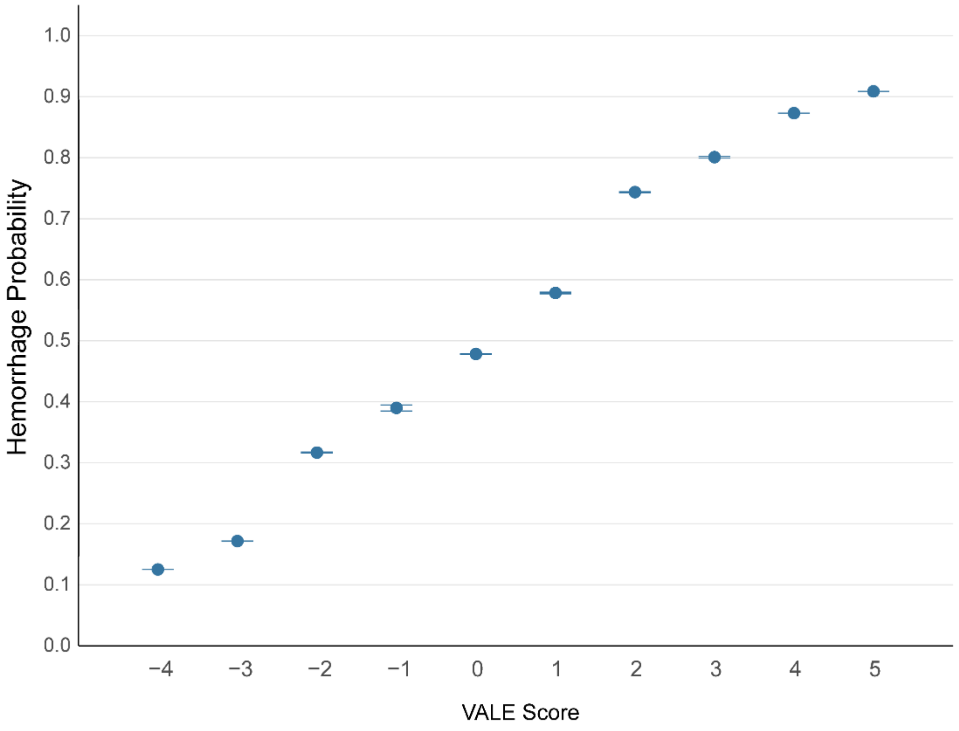

The probability of presentation with hemorrhage as predicted by the VALE Score for the derivation cohort. Supplement eMethods 2 showed how the probabilities were calculated.

© 2023 Chen Y et al. *JAMA Network Open*.

eTable 5. Subgroup Analysis of VALE Score in the Conservative Treatment Validation Cohort According to the Retrospective and Prospective Nature of the Survival Data

	Sample size	AUC	Specificity	Sensitivity	Accuracy	NPV
Complete cohort	1028	0.729 (0.645-0.813)	0.792 (0.769-0.819)	0.556 (0.389-0.722)	0.785 (0.761-0.809)	0.980 (0.973-0.987)
Prospective cohort	235	0.725 (0.599-0.851)	0.784 (0.729-0.839)	0.529 (0.294-0.765)	0.766 (0.715-0.817)	0.955 (0.934-0.977)
Retrospective cohort	793	0.734 (0.618-0.850)	0.795 (0.766-0.822)	0.579 (0.368-0.790)	0.789 (0.762-0.817)	0.987 (0.981-0.994)

Abbreviations: AUC, area under the curve; NPV, negative predictive value.

© 2023 Chen Y et al. *JAMA Network Open*.

eFigure 3. Illustration Cases for the VALE Score

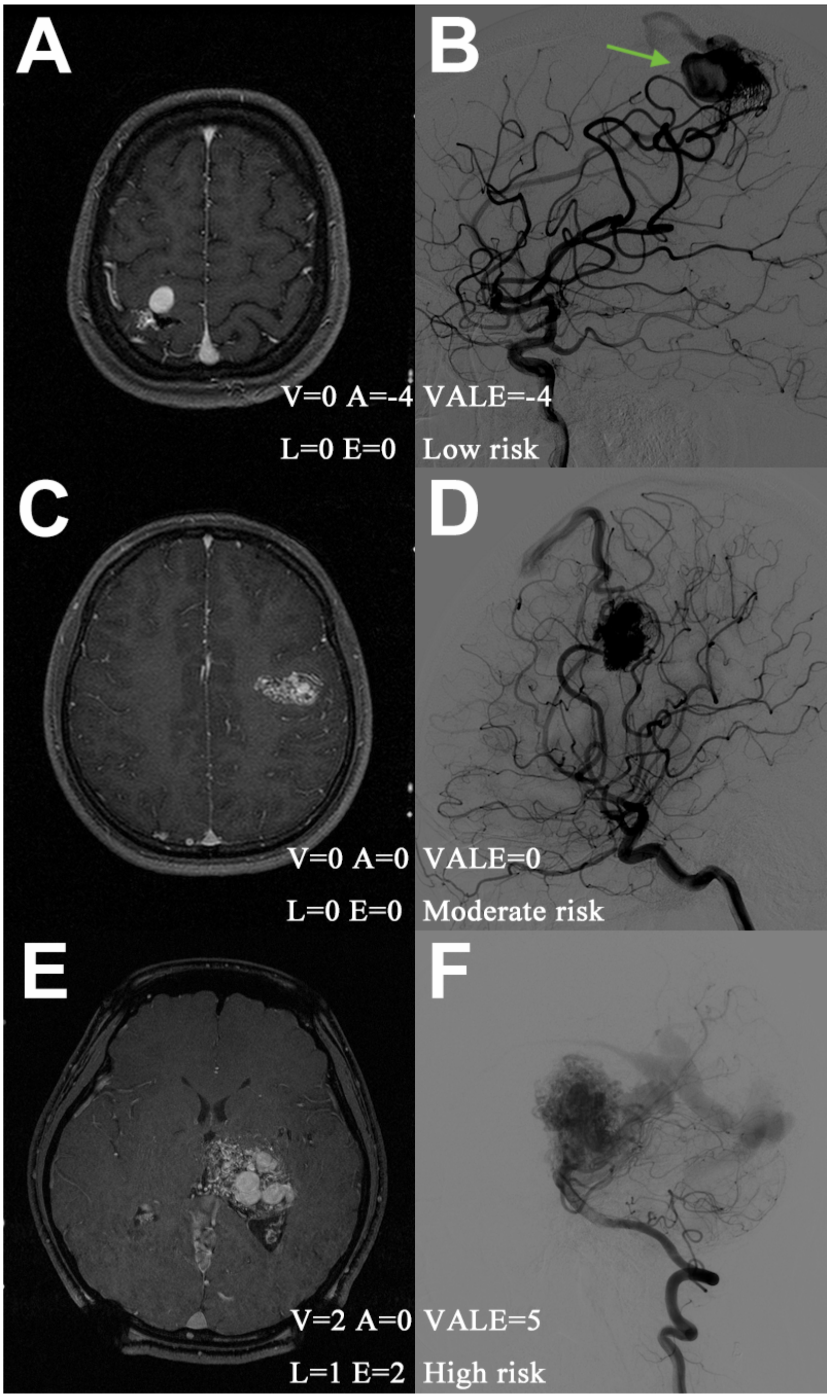

A-B, One unruptured AVM patient presenting with epilepsy was assessed as low-risk group by VALE score (Ventricular system involvement=0, venous Aneurysm=−4, deep Location=0, Exclusive deep drainage=0, VALE=−4), and had no subsequent hemorrhage within 8.75 years of follow-up after diagnosis (venous aneurysm: green arrow); C-D, One unruptured AVM patient presenting with dull headache was assessed as moderate-risk group (Ventricular system involvement=0, venous Aneurysm=0, deep Location=0, Exclusive deep drainage=0, VALE=0) by VALE score, and had no subsequent hemorrhage during 6.25 years of follow-up after diagnosis; E-F, A patient with unruptured AVM presenting with right limb weakness was assessed as a high-risk group by VALE score (Ventricular system involvement=2, venous Aneurysm=0, deep Location=1, Exclusive deep drainage=2, VALE=5), who had first hemorrhage 8.17 years after diagnosis and repeated rupture 3 times in the following 5 years.

© 2023 Chen Y et al. *JAMA Network Open*.

eTable 6. Univariable Analysis of Angiographic Features in Different Cohorts

Parameter	Derivation Cohort		External Validation Cohort		Conservative Cohort	
	OR (95% CI)	*P*	OR (95% CI)	*P*	HR (95% CI)	*P*
**Ventricular system involvement** [a]	4.11 (3.57-4.74)	<0.01	8.82 (5.43-14.34)	<0.01	4.04 (2.02-8.08)	<0.01
**Deep location** [a]	2.77 (2.33-3.30)	<0.01	2.63 (1.65-4.18)	<0.01	4.39 (2.22-8.70)	<0.01
Large nidus size	0.32 (0.28-0.37)	<0.01	0.67 (0.44-1.01)	0.06	1.06 (0.50-2.25)	0.89
Eloquent region	1.33 (1.16-1.52)	<0.01	1.32 (0.85-2.01)	0.21	4.01 (1.67-9.63)	<0.01
Single feeder	3.56 (3.01-4.20)	<0.01	1.72 (0.93-2.72)	0.02	0.69 (0.21-2.26)	0.54
Multiple source feeding	0.45 (0.39-0.53)	<0.01	0.73 (0.46-1.17)	0.19	1.71 (0.88-3.33)	0.11
**Perforating artery supply** [a]	1.72 (1.49-1.98)	<0.01	2.04 (1.32-3.13)	<0.01	3.09 (1.56-6.13)	<0.01
Aneurysm	1.37 (1.14-1.65)	<0.01	1.77 (1.04-3.00)	0.04	1.69 (0.83-3.45)	0.15
Diffuse nidus	2.80 (2.41-3.26)	<0.01	3.99 (2.41-6.60)	<0.01	0.92 (0.44-1.91)	0.82
Any deep drainage	2.02 (1.75-2.33)	<0.01	2.12 (1.39-3.24)	<0.01	2.33 (1.20-4.53)	0.01
**Exclusively deep drainage** [a]	4.51 (3.70-5.50)	<0.01	6.32 (3.48-11.46)	<0.01	3.60 (1.63-7.96)	<0.01
Venous stenosis	1.90 (1.56-2.32)	<0.01	5.45 (2.90-10.26)	<0.01	0.93 (0.28-3.04)	0.91
Venous aneurysm	0.15 (0.13-0.19)	<0.01	0.09 (0.05-0.15)	<0.01	0.42 (0.17-1.00)	0.05

Abbreviations: OR, odds ratio; HR, hazard ratio.

[a] Variables remains significant across three cohorts

© 2023 Chen Y et al. *JAMA Network Open*.

eReferences

1. Crawford PM, West CR, Chadwick DW, Shaw MD. Arteriovenous malformations of the brain: natural history in unoperated patients. *J Neurol Neurosurg Psychiatry*. 1986;49(1):1-10. doi:10.1136/jnnp.49.1.1
2. Mast H, Young WL, Koennecke HC, et al. Risk of spontaneous haemorrhage after diagnosis of cerebral arteriovenous malformation. *Lancet*. 1997;350(9084):1065-1068. doi:10.1016/s0140-6736(97)05390-7
3. Stapf C, Mast H, Sciacca RR, et al. Predictors of hemorrhage in patients with untreated brain arteriovenous malformation. *Neurology*. 2006;66(9):1350-1355. doi:10.1212/01.wnl.0000210524.68507.87
4. Kim H, Sidney S, McCulloch CE, et al. Racial/Ethnic differences in longitudinal risk of intracranial hemorrhage in brain arteriovenous malformation patients. *Stroke*. 2007;38(9):2430-2437. doi:10.1161/STROKEAHA.107.485573
5. Hernesniemi JA, Dashti R, Juvela S, Väärt K, Niemelä M, Laakso A. Natural history of brain arteriovenous malformations: a long-term follow-up study of risk of hemorrhage in 238 patients. *Neurosurgery*. 2008;63(5):823-829; discussion 829-831. doi:10.1227/01.NEU.0000330401.82582.5E
6. da Costa L, Wallace MC, Ter Brugge KG, O'Kelly C, Willinsky RA, Tymianski M. The natural history and predictive features of hemorrhage from brain arteriovenous malformations. *Stroke*. 2009;40(1):100-105. doi:10.1161/STROKEAHA.108.524678
7. Itoyama Y, Uemura S, Ushio Y, et al. Natural course of unoperated intracranial arteriovenous malformations: study of 50 cases. *J Neurosurg*. 1989;71(6):805-809. doi:10.3171/jns.1989.71.6.0805
8. Mine S, Hirai S, Ono J, Yamaura A. Risk factors for poor outcome of untreated arteriovenous malformation. *J Clin Neurosci*. 2000;7(6):503-506. doi:10.1054/jocn.2000.0743
9. Stefani MA, Porter PJ, terBrugge KG, Montanera W, Willinsky RA, Wallace MC. Large and deep brain arteriovenous malformations are associated with risk of future hemorrhage. *Stroke*. 2002;33(5):1220-1224. doi:10.1161/01.str.0000013738.53113.33
10. Yamada S, Takagi Y, Nozaki K, Kikuta K ichiro, Hashimoto N. Risk factors for subsequent hemorrhage in patients with cerebral arteriovenous malformations. *J Neurosurg*. 2007;107(5):965-972. doi:10.3171/JNS-07/11/0965
11. Graf CJ, Perret GE, Torner JC. Bleeding from cerebral arteriovenous malformations as part of their natural history. *J Neurosurg*. 1983;58(3):331-337. doi:10.3171/jns.1983.58.3.0331
12. Spetzler RF, Hargraves RW, McCormick PW, Zabramski JM, Flom RA, Zimmerman RS. Relationship of perfusion pressure and size to risk of hemorrhage from arteriovenous malformations. *J Neurosurg*. 1992;76(6):918-923. doi:10.3171/jns.1992.76.6.0918
13. Kim H, Al-Shahi Salman R, McCulloch CE, Stapf C, Young WL, MARS Coinvestigators. Untreated brain arteriovenous malformation: patient-level meta-analysis of hemorrhage predictors. *Neurology*. 2014;83(7):590-597. doi:10.1212/WNL.0000000000000688
14. Fults D, Kelly DL. Natural history of arteriovenous malformations of the brain: a clinical study. *Neurosurgery*. 1984;15(5):658-662. doi:10.1227/00006123-198411000-00003

© 2023 Chen Y et al. *JAMA Network Open*.
