# Supplementary material for: Development and Validation of a Scoring System for Hemorrhage Risk in Brain Arteriovenous Malformations
Source: JAMA Netw Open. 2023 Mar 1;6(3):e231070. doi: 10.1001/jamanetworkopen.2023.1070 (PMC9978947; doi:10.1001/jamanetworkopen.2023.1070)
Supplement: Supplement 2. — Data Sharing Statement [file jamanetwopen-e231070-s002.pdf]

Data Sharing Statement

Chen. Development and Validation of a Scoring System for Hemorrhage Risk in Brain Arteriovenous Malformations. *JAMA Netw Open*. Published March 01, 2023.
doi:10.1001/jamanetworkopen.2023.1070

Data
Data available: No

Additional Information
Explanation for why data not available: All original data are available upon reasonable request to the corresponding authors.
